# Editorial: Imaging Cerebrovascular Reactivity: Physiology, Physics and Therapy

**DOI:** 10.3389/fphys.2021.740792

**Published:** 2021-08-13

**Authors:** James Duffin, Molly G. Bright, Nicholas P. Blockley

**Affiliations:** ^1^Department of Anaesthesia and Pain Management, University of Toronto, Toronto, ON, Canada; ^2^Department of Physiology, University of Toronto, Toronto, ON, Canada; ^3^Thornhill Research Inc., Toronto, ON, Canada; ^4^Physical Therapy and Human Movement Sciences, Feinberg School of Medicine, Northwestern University, Chicago, IL, United States; ^5^Biomedical Engineering, McCormick School of Engineering and Applied Sciences, Northwestern University, Evanston, IL, United States; ^6^School of Life Sciences, University of Nottingham, Nottingham, United Kingdom

**Keywords:** cerebrovascular reactivity, cerebral blood flow, carbon dioxide, blood oxygenation level dependent signal, magnetic resonance imaging

## Cerebral Blood Flow

Brain tissue stores little energy and so requires a continuous supply of oxygen and glucose to maintain normal brain function and cell viability. With cerebral oxygen consumption accounting for ~20% of the total resting body oxygen consumption, this demand requires a continuous and well-regulated blood flow, and takes up to 15–20% of the total cardiac output (Helenius et al., 2003; Ito et al., 2004). Accordingly, several mechanisms are in place to maintain cerebral blood supply in the face of challenges such as variations in arterial blood pressure, hypoxemia, and vascular occlusions (Willie et al., 2014).

These include both the anatomical characteristics of the cerebral vasculature, such as the Circle of Willis, and physiological mechanisms. The latter includes the following mechanisms: (1) autoregulation to maintain flow against supply pressure changes (Tan and Taylor, 2014; Tzeng et al., 2014), (2) neurovascular coupling to increase flow in active regions (Attwell et al., 2011, 2016; Phillips et al., 2016), and (3) increased flow during hypoxemia (Duffin et al., 2020).

Other physiological factors also affect CBF. Acute changes in arterial blood gases (Willie et al., 2012), such as hypoxia (Cohen et al., 1967; Mardimae et al., 2012) and hypercapnia (Battisti-Charbonney et al., 2011), as well as decreases in hemoglobin (anemia) (Borzage et al., 2016) all impact cerebral blood flow (CBF). In the presence of hypoxia and anemia, vascular tone decreases to maintain adequate oxygen supply (Duffin et al., 2020). Long term changes in CBF occur in chronic anemia (Brown et al., 1985) including sickle cell anemia (Bush et al., 2016). These increases in flow required to maintain oxygen delivery, are accompanied by a multitude of adaptive changes orchestrated via the HIF 1 alpha pathway (Poellinger and Johnson, 2004) so that in the long term the cerebral vasculature remodels to provide larger diameter vessels to accommodate a higher CBF.

In normal physiological conditions, local demand for CBF is met by changes in the vascular resistance in parenchymal arterioles, with little variation in the global blood flow. The large pial arteries on the surface of the cortex contain multiple layers of vascular smooth muscle cells. These vessels branch into penetrating arterioles containing a single layer of vascular smooth muscle cells (Nishimura et al., 2007), and enter the cortical parenchyma where capillary pericytes may control the flow (Hall et al., 2014; Attwell et al., 2016). As detailed in this topic, the common effector pathway for controlling cerebral blood flow is changes in vascular diameter orchestrated by vascular smooth muscle (Duffin et al.). However, the mechanisms that drive this diameter change and subsequent CBF response may be complex and interconnected; as an example, CO_2_ may cause both direct vasodilation as well as indirect effects mediated by pressure changes (Battisti-Charbonney et al., 2011).

## Cerebrovascular Reactivity

Brain vascular health relates to a fundamental ability of the cerebrovascular system to match blood flow to tissue demand. Consequently, a measurement of the blood flow response to a vasodilator challenge constitutes a means of discerning the general health of the physiological regulators. This measurement is referred to as cerebrovascular reactivity (CVR). Numerous vasodilator stimuli exist, including pharmacological agents such as acetazolamide. At present, it is increasingly preferred to use a less invasive and more readily reversable dilatory stimulus such as hypercapnia: an increased arterial partial pressure of CO_2_ provides a strong global vasostimulation (Fierstra et al., 2013). The measurement of the CBF response can be made using transcranial Doppler (TCD) measurement of the velocities in large vessels, particularly the middle cerebral artery. However, the adoption of changes in blood-oxygen-level dependent (BOLD) signal measured with magnetic resonance imaging (MRI) have enabled the local responsiveness of the cerebrovasculature to be investigated with whole brain coverage. It should be noted, however, that there is no clear relationship between TCD- and BOLD- CVR measures (Burley et al.). The BOLD signal within an interrogated voxel arises from changes in flow in capillaries and venules and is therefore a proxy for the local tissue blood flow response. CVR is reported as the percent change in BOLD divided/normalized by the change in the partial pressure of CO_2_ due to the hypercapnic stimulus. This use of MRI measures to examine CBF changes on a voxelwise basis leads to the presentation of detailed CVR maps.

The development of CVR testing procedures has included aspects of both the vasodilatory CO_2_ stimulus as well as the detection of the resulting increase in cerebral blood flow with MRI (Sleight et al.). Endogenous CO_2_ changes that occur naturally with breathing have been considered as stimuli (Pinto et al.) as well as the CO_2_ and oxygen changes occurring during breath holding (Solis-Barquero et al.). The endogenous variations in CO_2_ and hence CVR, also interact with the measurement of intrinsic brain activity in resting state functional MRI, reviewed by Chen and Gauthier, and the effect of the removal of the respiratory variation in CO_2_ on resting state measures is demonstrated by McKetton et al..

Technical developments in the ability to control arterial CO_2_ via inspired gases have led to proposals for a standardization of a repeatable CO_2_ stimulus (Fisher and Mikulis) and experience with this methodology is described in Sobczyk et al. as well as its reproducibility across different MRI scanners by Sobczyk et al..

## Cerebrovascular Reactivity Data Analysis

Not only have CVR testing procedures matured but substantial advances have been made in the acquisition and analysis of the MRI data used to capture the CBF response. While BOLD contrast has been usually chosen as the surrogate measure of CBF for CVR measurement, other MRI sequences can also be used. A pseudocontinuous arterial spin labeling (pCASL) sequence combined with other adjustments was used to evaluate CVR in the study by Solis-Barquero et al. and investigations into the use of different MRI sequences has suggested improvements in CVR measurement (Cohen et al.). A comparison between CVR measurements at 1.5 and 3 Tesla showed that 3 Tesla MRI may reduce variance in CVR magnitude (Stringer et al.), and the use of ultra-high field MRI (Champagne and Bhogal) has generated insights into the temporal aspects of CVR. With the application of intensive processing, corrections for partial averaging, when the BOLD signal in a voxel originates from a mixture of tissue types, can be made as presented by Poublanc et al.

## Using CVR to Assess Vascular Health

One aspect of CVR measured using CO_2_ that pertains to its use in assessing vascular health is that the effect of CO_2_ is a global one, vasodilating all smooth muscle controlling cerebral blood flow. With BOLD MRI measuring CBF at a voxelwise spatial resolution the entire brain can be mapped. This aspect contrasts with typical task-activation functional MRI measurement where only particular regions are activated, and CBF increases via neurovascular coupling. With a global CO_2_ stimulus, flow changes are not only due to vasodilation but also to changes in the redistribution of local perfusion pressure brought about by the general increase in flow that increases the pressure loss in the major cerebral arteries (Faraci et al., 1987). An uneven distribution of the consequent local perfusion pressure decrease can lead to a CBF decrease in some regions where the cerebrovascular response to CO_2_ is lacking. This phenomenon is known as cerebrovascular steal, as though the healthy vasodilating voxels have stolen the flow from their unhealthy colleagues (Brawley, 1968).

The use of CVR as a clinically useful metric is slowly gaining recognition, with the transfer of research findings into clinical practice. CVR analysis was initially applied to measure the extent of steno-occlusive disease, but, as the papers presented in this Research Topic illustrate, CVR measures have applicability to a wide range of cerebrovascular pathologies, from assessing the impairment of CVR in sickle cell disease (Afzali-Hashemi et al.) as well as in chronic traumatic brain injury, where CVR relates to post-traumatic headache severity (Amyot et al.).

The challenges to its use as an effective diagnostic and prognostic tool in treatment planning and guidance, as well as the physiological mechanisms that lead to impaired CVR in aging and disease are reviewed by Krishnamurthy et al. That CVR may play an important role in cognitive decline is now recognized, and the relationship between BOLD-based assessments of CVR and cognition, and how altered CVR in disease and in normal physiology are associated with cognition are reviewed in Williams et al. The Heart and Brain Study protocol and objectives described by Suri et al. combines several measures including both CVR and cognitive testing. Experiments measuring the relationship between CVR and cognition in a study population at risk of cognitive decline demonstrate that while resting CBF was unrelated to cognitive scores, CVR was related to lower scores (Kim et al.).

Obstruction of an artery, either by thrombus or embolus, is the commonest cause for local ischemic damage. Brain tissue deprived of blood supply undergoes necrosis or infarction (stroke) (Victor et al., 2000). Steno-occlusive disease pathologies vary from extracranial or intracranial focal stenoses, to more progressive vasculopathies that affect multiple vessels. CVR assessment of these pathologies are complicated by the compensatory changes to mitigate ischemia such as downstream regional vasodilation and the development of collateral vasculature (Liebeskind, 2003). Consequently, the clinical appearance of steno-occlusive disease depends on the time course of the occlusion, its location, extent, and the availability of recruitable collateral circulation. It is the latter factor, more than the degree of local vascular impairment, that determines the risk of stroke (Ben Hassen et al., 2019).

Indeed, if recruitable collateral flow is available present on the side of carotid artery stenosis, there is a positive CBF response to hypercapnia with an increase in BOLD signal (Sobczyk et al.). Such a positive CVR has the lowest correlation with the risk of stroke regardless of the degree of stenosis (Reinhard et al., 2014). Wallerian degeneration and diaschisis are considered separate remote entities following ischemic stroke, and (van Niftrik et al.) show a strong association between Wallerian degeneration and ipsilateral thalamic diaschisis, indicating a structural pathophysiological relationship. There is also increasing evidence that impairments of cerebrovascular function may contribute to early neuronal cell loss in Huntington's disease, with preliminary CVR findings supporting that view (Chan et al.).

## Conclusion

We, the editors of this CVR Research Topic, hope that readers will benefit from the collection of articles presented. It is our conviction that CVR will prove to be ever more useful in the assessment of cerebrovascular disease and will see continued progress toward its adoption to clinical practice.

## Author Contributions

All authors listed have made a substantial, direct and intellectual contribution to the work, and approved it for publication.

## Conflict of Interest

JD was employed by company Thornhill Research Inc. The remaining authors declare that the research was conducted in the absence of any commercial or financial relationships that could be construed as a potential conflict of interest.

## Publisher's Note

All claims expressed in this article are solely those of the authors and do not necessarily represent those of their affiliated organizations, or those of the publisher, the editors and the reviewers. Any product that may be evaluated in this article, or claim that may be made by its manufacturer, is not guaranteed or endorsed by the publisher.
